# Supplementary material for: Modeling glioblastoma heterogeneity as a dynamic network of cell states
Source: Mol Syst Biol. 2021 Sep 16;17(9):e10105. doi: 10.15252/msb.202010105 (PMC8444284; doi:10.15252/msb.202010105)
Supplement: Supplementary file 6 — Source Data for Figure 5 [file MSB-17-e10105-s004.zip › Figure5A_sourcedata/GSEA_3071/nosigdb_stateVI.GseaPreranked.1622795169056/IVY_CTPAN.html]

Details for gene set IVY\_CTPAN[GSEA]

|  || Dataset | state63071 |
| Phenotype | NoPhenotypeAvailable |
| Upregulated in class | na\_pos |
| GeneSet | IVY\_CTPAN |
| Enrichment Score (ES) | 0.3306099 |
| Normalized Enrichment Score (NES) | 2.20663 |
| Nominal p-value | 0.0 |
| FDR q-value | 0.002870443 |
| FWER p-Value | 0.012 |
Table: GSEA Results Summary

  

Fig 1: Enrichment plot: IVY\_CTPAN      
 Profile of the Running ES Score & Positions of GeneSet Members on the Rank Ordered List

  

| PROBE | GENE SYMBOL | GENE\_TITLE | RANK IN GENE LIST | RANK METRIC SCORE | RUNNING ES | CORE ENRICHMENT || 1 | SERPINE1 |  |  | 0 | 1.673 | 0.0363 | Yes |
| 2 | CAV1 |  |  | 13 | 1.087 | 0.0566 | Yes |
| 3 | DDIT3 |  |  | 14 | 1.086 | 0.0802 | Yes |
| 4 | LOX |  |  | 29 | 0.968 | 0.0973 | Yes |
| 5 | RND3 |  |  | 49 | 0.866 | 0.1108 | Yes |
| 6 | PTX3 |  |  | 56 | 0.860 | 0.1277 | Yes |
| 7 | MT2A |  |  | 58 | 0.859 | 0.1461 | Yes |
| 8 | SNHG12 |  |  | 59 | 0.851 | 0.1646 | Yes |
| 9 | GLIPR1 |  |  | 81 | 0.781 | 0.1757 | Yes |
| 10 | GADD45B |  |  | 90 | 0.758 | 0.1899 | Yes |
| 11 | AKAP12 |  |  | 92 | 0.743 | 0.2058 | Yes |
| 12 | PPP1R15A |  |  | 98 | 0.738 | 0.2204 | Yes |
| 13 | IGFBP5 |  |  | 145 | 0.671 | 0.2221 | Yes |
| 14 | SNHG1 |  |  | 153 | 0.661 | 0.2345 | Yes |
| 15 | PYGL |  |  | 177 | 0.622 | 0.2415 | Yes |
| 16 | BIRC3 |  |  | 191 | 0.608 | 0.2511 | Yes |
| 17 | LDHA |  |  | 197 | 0.604 | 0.2628 | Yes |
| 18 | GADD45A |  |  | 200 | 0.603 | 0.2754 | Yes |
| 19 | PLP2 |  |  | 205 | 0.600 | 0.2873 | Yes |
| 20 | MAFF |  |  | 253 | 0.562 | 0.2863 | Yes |
| 21 | JUN |  |  | 321 | 0.527 | 0.2790 | Yes |
| 22 | MKNK2 |  |  | 326 | 0.524 | 0.2893 | Yes |
| 23 | CXCL3 |  |  | 359 | 0.508 | 0.2913 | Yes |
| 24 | GPRC5A |  |  | 371 | 0.502 | 0.2992 | Yes |
| 25 | MIR100HG |  |  | 374 | 0.501 | 0.3095 | Yes |
| 26 | CYR61 |  |  | 377 | 0.498 | 0.3197 | Yes |
| 27 | TRIB3 |  |  | 387 | 0.495 | 0.3280 | Yes |
| 28 | ATF3 |  |  | 416 | 0.483 | 0.3306 | Yes |
| 29 | ARL4C |  |  | 666 | 0.408 | 0.2697 | No |
| 30 | SYTL2 |  |  | 707 | 0.395 | 0.2671 | No |
| 31 | EFEMP1 |  |  | 763 | 0.384 | 0.2601 | No |
| 32 | SOCS3 |  |  | 775 | 0.381 | 0.2652 | No |
| 33 | MT1X |  |  | 867 | 0.366 | 0.2477 | No |
| 34 | VIM |  |  | 897 | 0.361 | 0.2474 | No |
| 35 | NFIL3 |  |  | 956 | 0.352 | 0.2388 | No |
| 36 | SDC4 |  |  | 1013 | 0.343 | 0.2306 | No |
| 37 | PGK1 |  |  | 1099 | 0.331 | 0.2140 | No |
| 38 | ADM |  |  | 1185 | 0.318 | 0.1971 | No |
| 39 | CEBPB |  |  | 1389 | 0.293 | 0.1466 | No |
| 40 | MIR155HG |  |  | 1394 | 0.292 | 0.1518 | No |
| 41 | SEC61G |  |  | 1435 | 0.287 | 0.1468 | No |
| 42 | TMEM158 |  |  | 1466 | 0.283 | 0.1446 | No |
| 43 | SESN2 |  |  | 1541 | 0.275 | 0.1298 | No |
| 44 | SLC2A1 |  |  | 1606 | 0.268 | 0.1177 | No |
| 45 | PDK1 |  |  | 1609 | 0.268 | 0.1230 | No |
| 46 | PFKFB4 |  |  | 1751 | 0.255 | 0.0891 | No |
| 47 | BNIP3 |  |  | 1778 | 0.253 | 0.0873 | No |
| 48 | HAS2 |  |  | 2025 | -0.282 | 0.0245 | No |
| 49 | VEGFA |  |  | 2104 | -0.298 | 0.0091 | No |
| 50 | ATP13A3 |  |  | 2318 | -0.336 | -0.0432 | No |
| 51 | H19 |  |  | 2503 | -0.379 | -0.0865 | No |
| 52 | FAM20C |  |  | 2610 | -0.403 | -0.1074 | No |
| 53 | DOK5 |  |  | 2799 | -0.457 | -0.1502 | No |
| 54 | WEE1 |  |  | 2974 | -0.527 | -0.1874 | No |
| 55 | ID2 |  |  | 3026 | -0.554 | -0.1897 | No |
| 56 | RDH10 |  |  | 3091 | -0.581 | -0.1950 | No |
| 57 | WSB1 |  |  | 3130 | -0.608 | -0.1925 | No |
| 58 | CXCR4 |  |  | 3302 | -0.759 | -0.2238 | No |
| 59 | NUPR1 |  |  | 3366 | -0.835 | -0.2233 | No |
| 60 | TGIF1 |  |  | 3380 | -0.860 | -0.2083 | No |
| 61 | TRIM9 |  |  | 3442 | -0.985 | -0.2040 | No |
| 62 | FOS |  |  | 3476 | -1.056 | -0.1903 | No |
| 63 | SPP1 |  |  | 3541 | -1.286 | -0.1803 | No |
| 64 | IL1RAP |  |  | 3551 | -1.364 | -0.1532 | No |
| 65 | PDPN |  |  | 3580 | -1.590 | -0.1265 | No |
| 66 | TNC |  |  | 3586 | -1.618 | -0.0928 | No |
| 67 | IGFBP3 |  |  | 3588 | -1.623 | -0.0578 | No |
| 68 | IGFBP2 |  |  | 3634 | -3.295 | 0.0011 | No |
Table: GSEA details [plain text format]

  

Fig 2: IVY\_CTPAN: Random ES distribution      
 Gene set null distribution of ES for **IVY\_CTPAN**
